# E2 Regulates Epigenetic Signature on Neuroglobin Enhancer-Promoter in Neuronal Cells

**DOI:** 10.3389/fncel.2016.00147

**Published:** 2016-06-01

**Authors:** Michela Guglielmotto, Stefania Reineri, Andrea Iannello, Giulio Ferrero, Ludovica Vanzan, Valentina Miano, Laura Ricci, Elena Tamagno, Michele De Bortoli, Santina Cutrupi

**Affiliations:** ^1^Neuroscience Institute of the Cavalieri Ottolenghi Foundation (NICO), University of TurinTurin, Italy; ^2^Department Neurosciences, University of TurinTurin, Italy; ^3^Center for Molecular Systems Biology, University of TurinOrbassano, Turin, Italy; ^4^Department of Clinical and Biological Sciences, University of TurinOrbassano, Turin, Italy; ^5^Department of Computer Science, University of TurinTurin, Italy

**Keywords:** neuroglobin, estrogen receptor, chromatin remodeling, genomic regions, epigenetic regulation

## Abstract

Estrogens are neuroprotective factors in several neurological diseases. Neuroglobin (NGB) is one of the estrogen target genes involved in neuroprotection, but little is known about its transcriptional regulation. Estrogen genomic pathway in gene expression regulation is mediated by estrogen receptors (ERα and ERβ) that bind to specific regulatory genomic regions. We focused our attention on 17β-estradiol (E2)-induced NGB expression in human differentiated neuronal cell lines (SK-N-BE and NT-2). Previously, using bioinformatics analysis we identified a putative enhancer in the first intron of NGB locus. Therefore, we observed that E2 increased the enrichment of the H3K4me3 epigenetic marks at the promoter and of the H3K4me1 and H3K27Ac at the intron enhancer. In these NGB regulatory regions, we found estrogen receptor alpha (ERα) binding suggesting that ERα may mediate chromatin remodeling to induce NGB expression upon E2 treatment. Altogether our data show that NGB expression is regulated by ERα binding on genomic regulatory regions supporting hormone therapy applications for the neuroprotection against neurodegenerative diseases.

## Introduction

17β-estradiol (E2) has neuroprotective activity in several models of neurodegenerative disorders, such as multiple sclerosis, ischemia, stroke and Alzheimer’s disease (AD; Arevalo et al., 2015). E2 genomic signaling is mediated by estrogen receptor alpha (ERα) and beta (ERβ), nuclear transcription factor receptors bound to regulatory genomic regions, known as promoters and enhancers. Furthermore, ERs form transcriptional complexes with several transcription factors and cofactors making long range interactions between promoter and enhancer of target genes (Welboren et al., 2009). ERs co-regulators contain chromatin remodeler factors, such as histone modifications enzymes. Chromatin dynamic, linked to epigenetic modifications, is a molecular mechanism for gene expression activation or repression (Magnani and Lupien, 2014; Heinz et al., 2015).

Epigenetic changes under E2 control have an impact on memory consolidation and energy expenditure in hypothalamus (Zhao et al., 2010; Frick et al., 2011; Frank et al., 2014; López and Tena-Sempere, 2015). E2 induces an increased expression of chromatin remodeling enzymes, such as histone deacetylases, demethylases and acetyltransferases (Zhao et al., 2010, 2012). In dorsal hippocampus, E2 regulates histone 3 acetylation on the brain-derived neurotrophic factor (BDNF) promoter (Fortress et al., 2014) and ERα, but not ERβ loss, induces impaired spatial memory in mice (Bean et al., 2014). In hypothalamus, E2 modulates energy homeostasis through several pathways, including transcriptional regulation of key genes in brain metabolism, such as oxytocin receptor, progesterone receptor (Gagnidze et al., 2013), leptin receptor (Bennett et al., 1999; Lindell et al., 2001) and pro-opiomelanocortin (Pelletier et al., 2007).

One target protein that increases upon E2 treatment is Neuroglobin (NGB), an oxygen-binding protein that acts as scavenger of reactive oxygen and nitrogen species. NGB plays a neuroprotective action against oxidative damage, after stroke injury and in AD models. E2 increases NGB levels in a dose-dependent manner (1–10 nM). This effect is reached in 1 h treatment and is maintained for 24 h in human neuroblastoma cell lines and in mouse primary hippocampal neurons. The E2 effect on NGB expression is evident also in primary astrocytes (De Marinis et al., 2011, 2013a). Neuronal hypoxia and cerebral ischemia increase NGB levels that protect against neurodegeneration because NGB silencing induces neuron apoptosis (Greenberg et al., 2008). E2 protects H_2_O_2_-induced apoptosis in neurons (De Marinis et al., 2011). NGB is overexpressed in early stage of AD and forms a complex with amyloid-β peptide (Sun et al., 2013; Seal et al., 2015).

NGB modulation upon E2 treatment is mediated by ERβ as demonstrated in human neuroblastoma cell line (SK-N-BE), in mouse primary hippocampal neurons, mouse astrocytes and several cancer cell lines. ERβ signaling is mediated by membrane-associated proteins and has an effect on mitochondria as apoptosis protection (De Marinis et al., 2011, 2013b; Fiocchetti et al., 2014, 2015). Although these are well assessed data, the question whether ERα may exert a direct effect on NGB transcription has not been worked out in detail, therefore we addressed this question in the present work using SK-N-BE and NT-2 differentiated cells as neuronal-like *in vitro* models. The comparison of two cell lines from different origin (SK-N-BE, human neuroblastoma cell lines, and NT-2, human embryonal carcinoma cell line) upon differentiation conditions represented models to study E2-induced NGB transcription regulation and find genomic regulatory regions.

Exploring ENCODE database, we found active histone marks and several transcription factors binding in the first intron of NGB locus of different neuronal cell lines (Cutrupi et al., 2014). Here, we found that E2 induced ERα binding on NGB locus and remodeled chromatin by changing epigenetic marks. We demonstrated that an intronic enhancer, bound by ERα upon E2 treatment, had active histone marks, suggesting that this intronic enhancer is an important regulatory region in NGB transcription regulation. These data showed a potential mechanism by which E2 may mediate neuroprotective action by NGB against neurotoxic stimuli opening the way to hormone therapy for aging.

## Materials and Methods

### Cell Lines and Treatments

SK-N-BE, human neuroblastoma cell line, were maintained in RPMI (Roswell Park Memorial Institute) 1640 medium containing 2 mM glutamine and supplemented with 100 mL/L fetal bovine serum, 10 mL/L non-essential amino acids, and 10 mL/L antibiotic mixture (penicillin-streptomycin amphotericin). For differentiation, 2 × 10^6^ were plated in 75 cm^2^ culture flasks (Corning Costar, Sigma-Aldrich, U.S.A.) and treated with 10 μM retinoic acid (RA) for 10 days.

NT-2 cells, human embryonal carcinoma cell line, were maintained in Dulbecco’s modified Eagle’s medium (DMEM)/F12 (Sigma-Aldrich, U.S.A.), supplemented with 5% fetal bovine serum and 1% antibiotic mixture comprising penicillin-streptomycin-amphotericin, in a humidified atmosphere at 37°C with 5% CO_2_. For differentiation, 2 × 10^6^ cells were plated in 75 cm^2^ culture flasks (Corning Costar, Sigma-Aldrich, U.S.A.) and exposed to 10 μM RA for 5 weeks. Growth medium was changed three times a week. Cells were then replated and, 48 h after, mitotic inhibitors cytosine arabinoside (1 μM), fluorodeoxyuridine (10 μM) and uridine (10 μM) were added for 2 weeks to inhibit the division of non-neuronal cells. Experiments were performed 4–5 weeks after cessation of RA treatment. Cells were grown for 24 h in hormone-deprived medium, obtained from phenol red-free DMEM (31053–028, Life Technologies, U.S.A) supplemented with 5% charcoal-dextran-treated serum, and were treated with 17β-estradiol (E2; E2758–1G, Sigma-Aldrich, U.S.A.) at a final concentration of 10 nM.

### Real Time RT-PCR

Total RNA was isolated using the TRIzol reagent (Life Technologies, U.S.A.). Five hundred nanograms of total RNA was reverse transcribed using the RETROScript cDNA synthesis kit (Life Technologies, U.S.A.).

Real-time PCR was performed using 7900HT Fast Real Time PCR by Applied Biosystems, the iTaqTM Universal SYBRR Green Supermix (Biorad) a Bio-Rad iQ iCycler detection system with SYBR green fluorophore. A melting curve analysis was made after each run to ensure a single amplified product for every reaction. All reactions were run at least in triplicate for each sample. Primers to study NGB expression were as follows NGB-Forward: 5′-TGGAAGACCTGTCCTCACTG-3′ and NGB-Reverse: 5′-GAGCAGAGACTCACCCACTG-3′ (Zhang et al., 2011). Gene expression was normalized using specific amplification of 18S.

### Immunoblot Analysis

SK-N-BE and NT-2 neuronal cells, treated with the appropriate experimental conditions, were quickly placed on ice and washed with ice-cold PBS. Whole-cell extracts were prepared in ice-cold lysing buffer Protein concentration in the supernatant was quantified in triplicate by bicinchoninic acid (BCA) assay. For determination of NGB, 100 μg of cells lysates were separated in 15% gels, transferred onto nitrocellulose, and revealed respectively with antibody anti-NGB (Santa Cruz). Membranes were stripped and incubated with anti-actin antibody. Reactions were developed with enhanced chemiluminescence (ECL) system according with the manufacturer’s protocol (Amersham-Pharmacia, Biotech, Italia, Cologno Monzese, Italy). Densitometric analysis was performed by using a software program (Multi-analyst, version 1.1, Bio-Rad Laboratories, Segrate, Italy). NGB signal were normalized to those of corresponding actin, were expressed as arbitrary units of optical density and were means ± SD of three independent experiments.

### Chromatin Immunoprecipitation Assay (ChIP)

Differentiated SK-N-BE and NT-2 were treated with vehicle or E2 and incubated for 45 min and 1 h, after which they were treated with 1% formaldehyde in PBS for 10 min at room temperature on a platform shaker. The crosslinking reaction was stopped by adding glycine at a final concentration of 125 mM. Cells were rinsed twice with cold PBS before harvesting and crosslinked cells were then resuspended in Cell Lysis buffer (5 mM Pipes pH 8.0, 85 mM KCl and 0.5% NP-40). After a 10 min incubation on ice, nuclei were collected by centrifugation and resuspended in Nuclei Lysis buffer (50 mM Tris-HCl pH 8.1, 10 mM EDTA and 1% SDS). Cell lysates were incubated on ice for 10 min and then sonicated 10 times for 20 s at 30% amplitude (SonoPlus HD2070 sonicator). Small portion of sonicated chromatin (25 μl) was used to verify that the average size of DNA fragments was in the range of 250–500 bp. DNA/protein complexes (30 μg) were diluted in ImmunoPrecipitation buffer (0.01% SDS, 1.1% Triton X-100, 1.2 mM EDTA, 16.7 mM Tris-HCl pH 8.0 and 167 mM NaCl) to a final volume of 400 μl. Immunoprecipitations were performed by adding 1 μg of antibodies against human ERα H184 and HC20 (Santa Cruz Biotechnology, U.S.A.), H3K4me3 and H3K4me1 (Diagenode, Belgium), H3K27me3 and H3K27Ac (Active Motif, U.S.A) and IgG (Abcam, UK) and then incubating at 4°C overnight with rotation. Samples with IgG antibody were run in parallel as negative controls. The following day, 30 μl of 50% Protein A Sepharose^TM^ 4 Fast Flow (GE Healthcare, UK) slurry was added and incubated for 2 h at 4°C to capture the immune complexes. Proteins and DNA nonspecifically associated with the beads were removed by sequentially washing with low-salt buffer (0.1% SDS, 1% Triton X-100, 2 mM EDTA, 20 mM Tris-HCl pH 8.0 and 150 mM NaCl), high-salt buffer (0.1% SDS, 1% Triton X-100, 2 mM EDTA, 20 mM Tris-HCl, pH 8.0 and 500 mM NaCl), LiCl washing buffer (0.25 M LiCl; 1% deoxycholate sodium salt, 1 mM EDTA, 10 mM Tris-HCl pH 8.0 and 1% NP-40) and twice with Tris-EDTA buffer (10 mM Tris-HCl pH 8.0, 1 mM EDTA) at 4°C for 5 min each wash. The immunoprecipitated DNA–protein complexes were eluted from beads with 1% SDS in 0.1 M NaHCO_3_. Then the DNA–protein complexes were incubated with 0.2 M NaCl at 65°C overnight followed by proteinase K digestion for 1 h at 45°C to reverse cross-linking. After protein removal, DNA was purified by phenol/chloroform extraction followed by Et-OH precipitation.

Quantification of ChIP enriched DNA was performed by real-time PCR using iTaq Universal SYBR Green Supermix (Bio-Rad, U.S.A.). The enrichment of target sequences in the immunoprecipitated samples was normalized on input samples (1% of total chromatin used per IP) and expressed as enrichment of specific binding over the control unspecific IgG binding. Custom ChIP primers are: NGB promoter forward: 5′-GAGGCGACCAAATTCAACAC-3′, reverse: 5′-TGCAGAACGTAACTGACATCG-3′; NGB intronic enhancer forward: 5′-CAGCTTGGATGTAGTGCAGC-3′, reverse: 5′-TTCAGTTACCCGGTGAGACC-3′; GAPDH (Neg) forward: 5′-TACTAGCGGTTTTACGGGCG-3′, reverse: 5′-TCGAACAGGAGGAGCAGAGAGCGA-3′.

### Bioinformatic Analysis

The FANTOM5 datasets (http://fantom.gsc.riken.jp/5/) contains transcription start sites (TSSs) and their expression in human and mouse primary cells, tissues and cell lines using cap analysis of gene expression (CAGE) (Forrest et al., 2014). We searched for NGB and analyzed NGB locus using the Zenbu browser genomic tool (Severin et al., 2014). We selected the transcripts, enriched in NGB TSS and in the first intron, expression. For the evaluation of NGB expression level, the main CAGE peak attributed to the gene (p1@NGB) was considered. For the evaluation of the expression level of the RNAs transcribed at the intronic enhancer of NGB, a window of 100 bp focused on the cluster of CAGE peak (chr14:77,735,777-7,773,587, hg19 genome assembly) was selected. The 20 samples associated with the highest CAGE level were considered.

### Statistical Analysis

Statistics were carried out with the GraphPad Prism version 5.03 for Windows by 1-way ANOVA followed by Bonferroni *post hoc* test.

## Results

### NGB Locus in FANTOM5 Database

**Figure 1:**
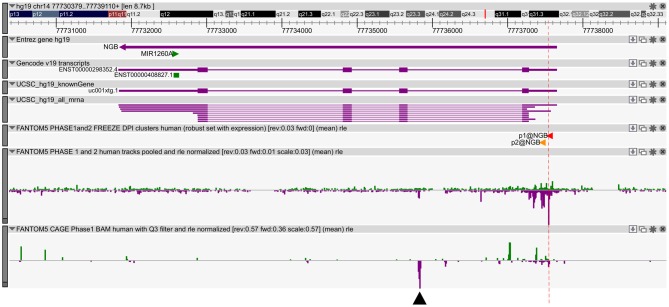
**Neuroglobin (NGB) locus and transcript expression in FANTOM5 database.** Zenbu Genome Browser of human NGB locus (chr14:77,730,566-77,739,297). In the first three lanes, the transcript annotations from Entrez, Gencode and UCSC database are reported. Sense transcripts are in green and antisense in purple. The red lane marks the high level of transcription start site (TSS) transcripts. Black arrow indicates antisense transcript in the first intron (chr14: 77,735,803-77,735,866).

**Figure 2:**
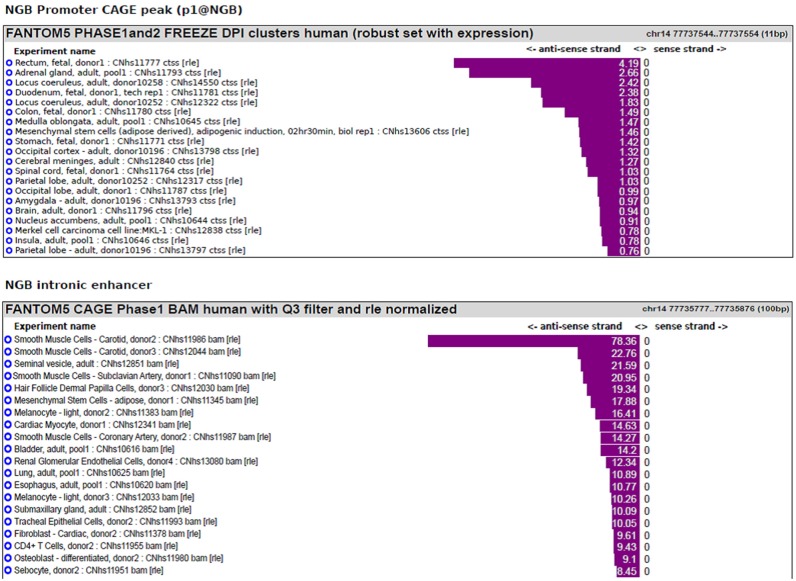
**NGB main transcript expression in FANTOM5 database.** Bar plot reporting the tissues associated with the highest level of transcripts annotated to the main NGB TSS (p1@NGB) or to the intronic enhancer.

We previously published bioinformatic analysis on NGB locus to identify putative regulatory regions. On this way, we explored a new FANTOM5 database, including CAGE experiments across over 900 human samples (Forrest et al., 2014). We found several transcripts in correspondence of the TSS and in the first intron of NGB gene (Figure 1). The first intronic transcript maps on the enhancer that we have previously predicted (chr14:77,735,963-77,736,462; hg19; Cutrupi et al., 2014). The presence of the transcripts in an enhancer indicates a site of non-coding RNA transcription and these transcripts may regulate gene expression. Therefore, this region may act as long distance genomic regulatory region for nearby genes (Ong and Corces, 2011). However, the expression data on transcripts stemming from the NGB promoter revealed high abundance in several brain regions, particularly in the *locus coeruleus*, *occipital cortex and lobe, amygdala* (Figure 2; NGB promoter CAGE peak). On the contrary, first intronic transcripts are highly expressed in non-neuronal tissues identifying this genomic region (Figure 2; NGB intronic enhancer).

### E2 Induces NGB Expression in SK-N-BE and NT-2 Differentiated Cells

**Figure 3:**
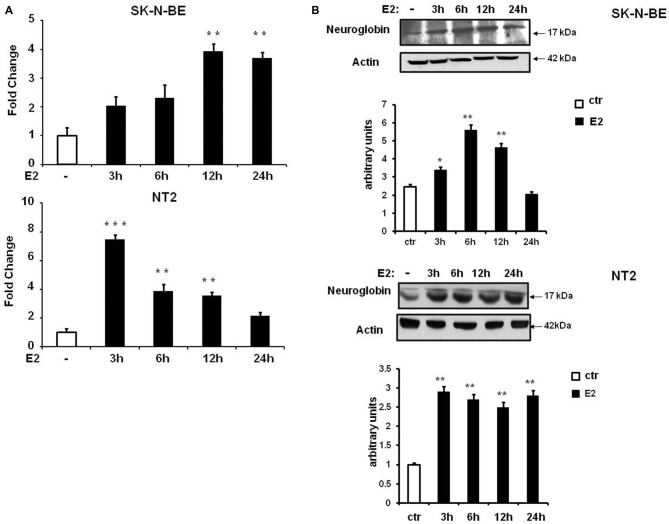
**NGB mRNA and protein expression in SK-N-BE and NT-2 differentiated neuroblastoma cells. (A)** Time-course of NGB mRNA after 10 nM 17β-estradiol (E2) treatment. Error bars are ± SEM of three independent biological replicates: *p-value < 0.05, **p-value < 0.01 and ***p-value < 0.001. The *p*-values were calculated with one-way analysis of variance (ANOVA) followed by Bonferroni *post hoc* test. **(B)** Time-course of NGB protein after 10 nM E2 treatment. In all cases diagram shows the mean normalized densitometry values.

E2-induced NGB protein expression in neuroblastoma cell lines, SK-N-BE, has been previously demonstrated (De Marinis et al., 2011) and, here we investigated SK-N-BE and NT-2 cells upon conditions leading to cell differentiation. Cells were plated and cultured in full medium, then maintained in hormone depleted medium for 24 h before being treated with 10 nM E2 and collected at different time-points (3, 6, 12 and 24 h). Cells grown in hormone depleted medium and treated 24 h with 10 nM Et-OH vehicle were used for comparison. By using quantitative RT-PCR we observed that E2 induced NGB mRNA expression with different kinetic in the two cell types of cells. SK-N-BE cells showed 4-fold increase of NGB mRNA after 12 h of E2 treatment (*F*_(4,15)_ = 7.7, *P* < 0.001), and NT-2 cells showed NGB mRNA level increase (~6 fold) after 3 h and is maintained until 12 h (*F*_(4,15)_ = 44.5, *P* < 0.0001; Figure 3A). We analyzed NGB protein level using Western blot: NGB protein increased 2-fold after 6 h of E2 treatment in SK-N-BE cells and 3-fold after 3 h in NT-2 cells (Figure 3B). NGB kinetic activation between mRNA and protein upon E2 treatment is different suggesting that E2 may regulate NGB by using multiple mechanisms.

### E2 Induces Chromatin Remodeling on NGB Locus in SK-N-BE and NT2 Differentiated Cells

**Figure 4:**
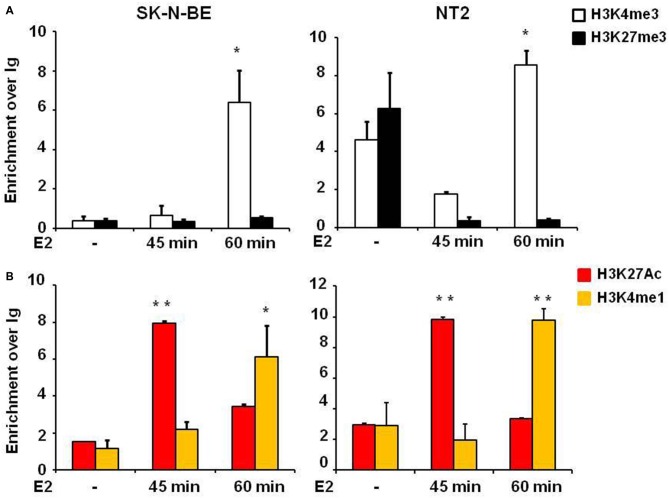
**E2 regulates histone modifications in regulatory genomic regions of** **differentiated SK-N-BE and NT-2 neuroblastoma cells. (A)** ChIP-qPCR analysis of H3K4me3 and H3K27me3 at the promoter of differentiated SK-N-BE and NT-2 cells, treated with 10 nM E2 for 45 min and 1 h. **(B)** ChIP-qPCR analysis of H3K4me1 and H3K27Ac at the intronic region differentiated SK-N-BE and NT-2 cells, treated with 10 nM E2 for 45 min and 1 h. Error bars are ± SEM of three independent biological replicates: *p-value < 0.05 and **p-value < 0.01. The *p*-values were calculated with 1-way ANOVA followed by Bonferroni *post hoc* test.

One step of transcription regulation for gene expression is chromatin remodeling. The dynamic changes of epigenetic modifications are associated with transcription regulation upon E2 treatment (Métivier et al., 2003). Histone marks define the specific states of chromatin: histone 3 lysine 4 trimethylation (H3K4me3) is a marker of active state corresponding to gene transcription activation in the promoter, histone 3 lysine 27 trimethylation (H3K27me3) is a marker of silenced state corresponding to gene transcription silencing, whereas histone 3 lysine 4 monomethylation (H3K4me1) and histone 3 lysine 27 acetylation (H3K27Ac) are markers of active enhancers. E2 induced the enrichment of H3K27Ac in the active enhancer (Hah et al., 2013). To address the underlying molecular mechanisms of how E2 regulates NGB expression we explored histone modifications at the NGB promoter and intronic enhancer using chromatin immunoprecipitation followed by quantitative RT-PCR (ChIP-qPCR). H3K4me3 enrichment was significantly increased on NGB promoter in both cell types after 1 h of E2 treatment (*F*_(2,9)_ = 11.6, *P* < 0.02 for SK-N-BE and *F*_(2,9)_ = 44.1, *P* < 0.002 for NT-2; Figure 4A). H3K27Ac (*F*_(2,9)_ = 71.4, *P* < 0.001 for SK-N-BE and *F*_(2,9)_ = 22.3, *P* < 0.002 for NT-2) and H3K4me1 (*F*_(2,9)_ = 15.2, *P* < 0.01 for SK-N-BE and *F*_(2,9)_ = 62.4, *P* < 0.001 for NT-2), increased after 45 min and 1 h of E2 treatment in the same manner for the two cell types (Figure 4B). Altogether these data suggest that E2 may regulate NGB transcription by inducing epigenetic remodeling of NGB promoter and intronic enhancer.

### ERα Binds to NGB Promoter and Intronic Enhancer upon E2 Treatment in SK-N-BE and NT-2 Differentiated Cells

**Figure 5:**
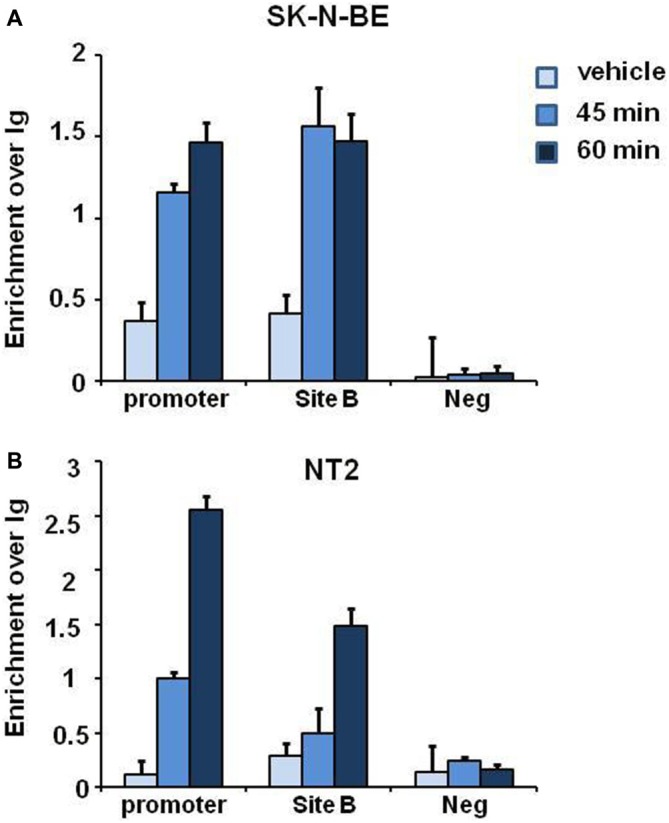
**E2 induces estrogen receptor alpha (ERα) association on promoter and intron regions of differentiated SK-N-BE and NT-2 neuroblastoma cells.** ChIP-qPCR analysis of ERα binding at the promoter, intronic enhancer (Site **B**) and negative control region, GAPDH, of differentiated SK-N-BE **(A)** and NT-2 **(B)** cells, treated with 10 nM E2 for 45 min and 1 h. Error bars are ± SEM of three independent biological replicates.

E2 induced histone modifications changes and coexpression of ERα and NGB in different brain regions suggest that E2 may regulate NGB expression by ERα. To verify whether ERα binds to NGB promoter and putative enhancer (chr14:77,735,963-77,736,462; hg19; Site B), we performed ChIP-qPCR experiments in SK-N-BE and NT-2 differentiated cells using ERα-specific antibodies. E2 induced ERα binding at NGB promoter and first intron enhancer after 1 h treatment (Figure 5). GAPDH promoter was used as negative control for ERα binding. These data suggest that E2-induced ERα binding on NGB regulatory regions may recruit co-regulators able to modify chromatin states for NGB transcription activation.

## Discussion

The present study identifies a novel regulatory domain in the first intron of NGB locus acting as enhancer. E2 induced binding of ERα and epigenetic modifications in this genomic region in concert with the promoter. Therefore, we describe ERα-mediated genomic pathway able to regulate NGB transcription upon E2 treatment.

E2 induces ERα recruitment preferentially to estrogen response element (ERE) regions. *In silico* analysis revealed the absence of a potential ERE in NGB promoter and intron enhancer. Sp1 and NF-kB transcription factor binding sites are present on NGB promoter and their binding is demonstrated in neuroblastoma cell lines (Liu et al., 2012). Therefore, ERα binding may be mediated by Sp1 and NF-kB (Welboren et al., 2009). Another transcription factor that mediates ERα binding is cAMP response element-binding protein (CREB), bound on mouse NGB promoter in N2a neuroblastoma cells (Liu et al., 2013). Exploring the ENCODE database, we found that several transcription factors may bind the NGB intronic enhancer supporting the hypothesis that an active transcriptional complex associated with the intronic enhancer may form an interaction loop with NGB promoter (Cutrupi et al., 2014). The FANTOM5 database showed that the first intron transcripts corresponded to predictive intronic enhancer. CAGE tags identify bidirectional capped RNAs that are overlapped with active histone marks for enhancers, H3K27Ac (Andersson et al., 2014). However, these first intron transcripts are expressed in smooth muscle where NGB expression is not yet detectable suggesting that the first intronic enhancer may be in active state in this specific tissue under basal conditions as a putative alternative TSS because there is a unidirectional transcript. In neuronal cells, the first intronic enhancer may shift from poised to active state after stimuli. In our neuronal models the intronic enhancer acquires active histone marks upon E2 treatment, even though there are not any transcripts in these specific cell types reported in FANTOM5 library. Therefore, the intronic enhancer may become active after E2 stimulus in neuronal models.

After gene co-expression analysis based on Allen Brain Atlas database (ABA), we found that ERα and NGB are highly co-expressed in hypothalamus (Cutrupi et al., 2014). Using ABA gene expression profiling analysis across specific brain regions, steroid hormone receptors co-expression is associated with cell-types-specific area. In particular, ERα is highly expressed in the specific regions of hypothalamus in according with estrogen-regulated genes, such as NGB (Mahfouz et al., 2016). Estrogen effects in hypothalamic energy homeostasis are largely mediated by ERα as demonstrated by ERα and ERβ knock out models (López and Tena-Sempere, 2015). In addition, NGB is also expressed in hippocampus in NGB-overexpressed transgenic mice, under hypoxia and AD (Raida et al., 2013; Sun et al., 2013) and both ERα and ERβ are associated with the same pathological conditions (Arevalo et al., 2015). Altogether these data showed that brain-region-specific molecular mechanisms may regulate NGB expression upon E2 treatment.

E2 regulates NGB expression through both genomic and non-genomic pathways as demonstrated in neuronal and non-neuronal cellular models. E2 enhances NGB protein level in neuronal cells and induces NGB localization in mitochondria where, after oxidative stress injury, NGB inhibits pro-apoptotic signaling by ERβ (De Marinis et al., 2013b). In mouse cortical astrocytes, ERβ mediates E2-induced NGB expression and in lipopolysaccharide (LPS)-induced pro-inflammatory signaling (De Marinis et al., 2013a). In cancer cell lines, ERα is overexpressed respect to ERβ and mediates NGB expression upon E2 treatment (Fiocchetti et al., 2014). In this work we used NT-2 differentiated cells where ERα is over-expressed in respect to ERβ, therefore ERα contribution to E2-induced chromatin remodeling at NGB locus may be predominant.

We found that E2 induced ERα binding to NGB promoter and intron enhancer, correlating with the dynamics of epigenetic modifications. E2 induced an increased level of active histone marks H3K4me3 at the NGB promoter and H3K4me1-H3K27Ac at the intronic enhancer. ERα is expressed in specific hypothalamus regions that are important in regulating energy homeostasis (Frank et al., 2014). The molecular mechanisms that occur in chromatin remodeling may be an ERα-induced recruitment of co-regulators with enzymatic activity to induce histone modifications at the regulatory regions (Green and Carroll, 2007). Several factors are described to form ERα complexes in breast cancer models, but little is known in nervous system. SRC-1 and SRC-2 cofactors, associated with histone acetyltransferase p300/CBP are expressed in specific hypothalamic neurons and regulated progesterone receptor expression isoforms (Apostolakis et al., 2002; Gagnidze et al., 2013; Acharya et al., 2015).

In addition, E2 regulated the expression of enzymes that carry out histone modifications: increased histone 3 acetylation and decreased histone deacetylase, HDAC2 and HDAC3, in hippocampus (Frick, 2015). In memory consolidation, histone modifications changes are described on BDNF (Fortress et al., 2014). In this context, E2-induced memory effects are mediated by non-genomic pathway where ERK and PI-3 Kinase activated CREB, which represents the transcription factor driving chromatin remodeling and regulation of gene expression (Frick, 2015).

In pathological conditions, E2-induced NGB may mediate an anti-apoptotic response (De Marinis et al., 2011). The decreased levels of E2 during menopause are associated with high risk of cognitive impairment, stroke and AD. E2 has a neuroprotective effect in several animal models of neurodegenerative diseases, such as ischemia, AD and experimental autoimmune encephalomyelitis (EAE; Arevalo et al., 2015). In addition, ERα interaction with neurofibrillary tangle and NGB-Aβ complexes suggest that proteins aggregates with ERα and NGB may inhibit their functions, therefore de-regulation impairs neuroprotective effect in AD (Sun et al., 2013; Seal et al., 2015; Wang et al., 2016). NGB is co-expressed with ERα in specific hypothalamic regions (Hundahl et al., 2008; Cutrupi et al., 2014). NGB is a heme-protein with oxidative protection function and forms a complex with cytochrome c in mitochondria (Yu et al., 2012). E2 regulated energy homeostasis acting on hypothalamic-specific regions and E2 decline during aging was associated with body weight increase and fat distribution (Fontana et al., 2015). Altogether, these data suggest that E2-induced NGB may regulate mitochondria function: control of glucose metabolism, ATP generation and neuronal apoptosis.

NGB is also expressed in hippocampus where ERα functions is important in memory formation, while ERβ decreased estrogen-target genes (Han et al., 2013; Cho et al., 2015; Frick, 2015). ERα knock out and ERα-overexpressed models demonstrated the central role in the memory function (Foster et al., 2008; Bean et al., 2014). ERα downregulation was associated with memory impairment during aging and menopause period where estrogens decline (Frick, 2015). Estrogens regulated neurogenesis in hippocampus and play a central role in neuroplasticity (Barha and Galea, 2010). Our work points attention on epigenetic modifications in genomic regulatory regions upon estrogen treatment, on the same way of epigenetic changes that occur during aging (Frick et al., 2011). Co-expression of ERα and NGB in several brain regions suggests that they may regulate different signaling important for memory formation in the hippocampus and for energy homeostasis in the hypothalamus. The data obtained for NT-2 and SK-N-BE differentiated cells may suggest that transcriptional complex composition and environment regulate NGB expression upon E2 treatment. Therefore, these data in the cellular models support the further experiments in neurodegenerative mice models to investigate which is the epigenetic context advantageous for hormone therapy.

## Author Contributions

MG, SR, AI, LV, GF, VM, LR, ET and MDB contribute to: Substantial contributions to the conception or design of the work; or the acquisition, analysis, or interpretation of data for the work; and drafting the work or revising it critically for important intellectual content; and final approval of the version to be published; and agreement to be accountable for all aspects of the work in ensuring that questions related to the accuracy or integrity of any part of the work are appropriately investigated and resolved.

## Conflict of Interest Statement

The authors declare that the research was conducted in the absence of any commercial or financial relationships that could be construed as a potential conflict of interest.
